# Metastatic Cardiac Tumor Presenting as an Anteroseptal ST-Segment Elevation Myocardial Infarction in a Young Male

**DOI:** 10.7759/cureus.13981

**Published:** 2021-03-18

**Authors:** John-Henry L Dean, Arjun G Kalra, Shayef Gabasha, Rosco Gore

**Affiliations:** 1 Department of Internal Medicine, Brooke Army Medical Center, San Antonio, USA; 2 Department of Cardiology, Brooke Army Medical Center, San Antonio, USA

**Keywords:** st-segment elevation myocardial infarction (stemi), bone and soft-tissue sarcoma, metastatic tumor to the heart, pericardial tamponade

## Abstract

In the appropriate clinical context, ST-segment elevation on electrocardiogram (ECG) necessitates prompt evaluation for coronary artery occlusion requiring reperfusion with percutaneous coronary intervention. Conversely, the etiology of ST-segment elevation may be representative of an alternative diagnosis other than myocardial infarction. We report the case of a patient with a history of primary bone sarcoma who presented for further evaluation of a large pericardial effusion identified on an outpatient echocardiogram and was found to have ST-segment elevation on ECG in the absence of any cardiopulmonary symptoms. The ECG abnormalities were attributed to a likely persistent current of injury resulting from a mass in the interventricular septum, likely representative of a metastatic lesion of his known malignancy. This case highlights the importance of maintaining a broad differential for ST-segment elevation, particularly in patients without symptoms of angina and those with a history of aggressive or relapsing cancer to minimize the morbidity and mortality of invasive procedures.

## Introduction

Elevation of the ST-segment on electrocardiogram (ECG) typically generates a list of potential diagnoses comprising common etiologies (e.g., ST-segment elevation myocardial infarction [STEMI], early repolarization, pericarditis, and ST-segment elevation secondary to a primary depolarization abnormality such as left ventricular hypertrophy/left bundle branch block or pre-excitation), as well as less common etiologies (e.g., Brugada syndrome, hyperkalemia, and pulmonary embolism) [1]. Cardiac neoplasms represent a rare clinical entity [2,3], and as such, are infrequently considered in the differential diagnosis of ST-segment elevation. Several case reports have been published on ST-segment changes in the context of cardiac tumors, though the precise pathophysiological mechanism behind the ECG changes continues to be debated. Suggested hypotheses for these changes include embolization of tumor fragments to coronary arteries, external compression of coronary arteries, distension of cardiac muscle fibers, peri-tumor inflammatory reactions, and electrolyte transfer from necrotic tumor tissue to adjacent myocardium [4,5]. Regardless of the particular mechanism involved, most documented cases of ST-segment elevation in the setting of cardiac tumors often appear to present with chest pain/discomfort or clinical signs of developing heart failure [4,5]. Here, however, we describe the case of a patient who presented with ST-segment elevation in the absence of any cardiopulmonary symptoms.

## Case presentation

**Figure 1:**
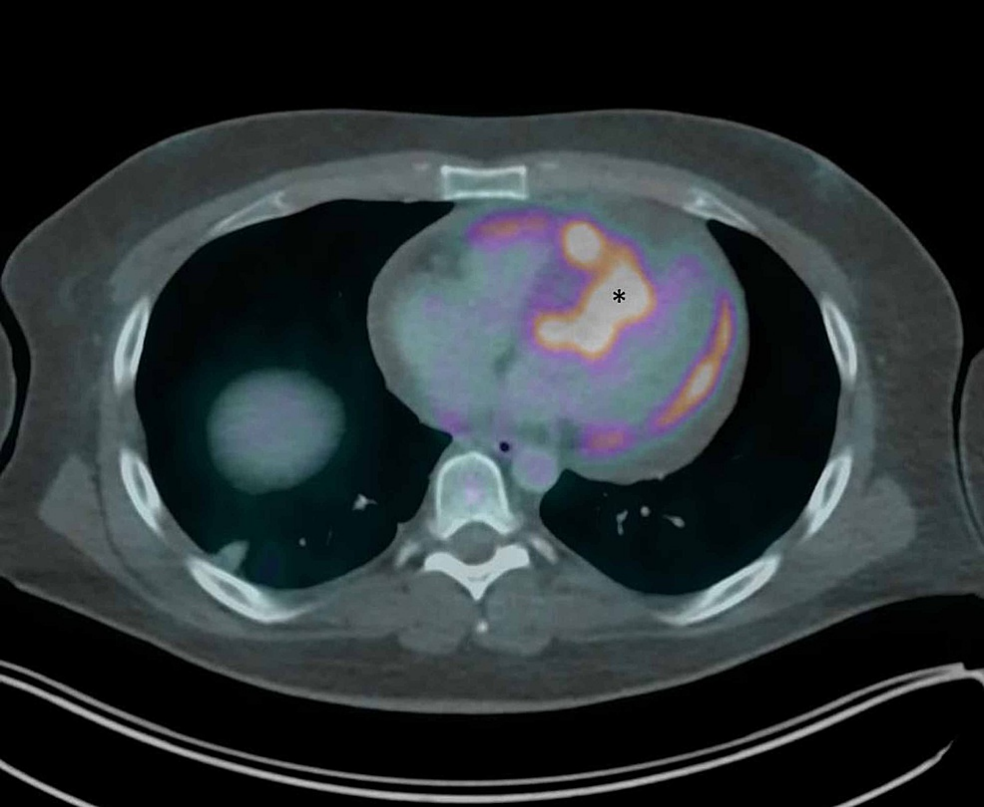
PET scan demonstrating area of increased radiotracer uptake and metabolic activity (asterisk) at the site of the cardiac tumor in the interventricular septum. PET, positron emission tomography

**Figure 2:**
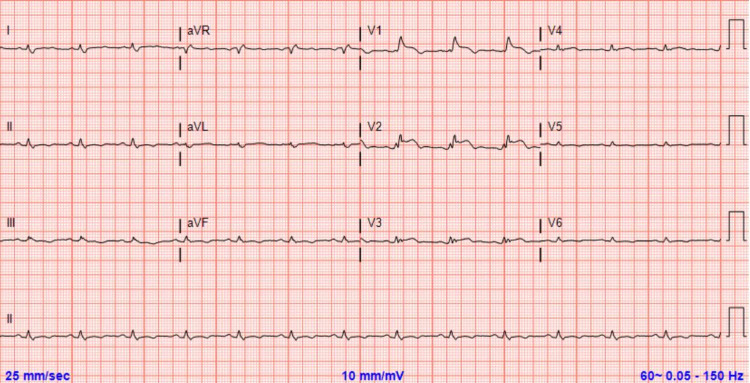
Initial ECG showing sinus rhythm, low voltage, and ST-segment elevation in anteroseptal precordial leads. ECG, electrocardiogram

A 28-year-old, active-duty military male with relapsed, refractory, metastatic, malignant, giant-cell-rich, primary bone sarcoma presented to the Emergency Department (ED) by cardiology instruction for evaluation of a large pericardial effusion with possible early tamponade physiology that was identified on an outpatient transthoracic echocardiogram (TTE). The echocardiogram was performed to further evaluate findings from a positron emission tomography (PET) scan completed three days prior that demonstrated findings concerning for cardiac metastasis to the interventricular septum with extension into the right ventricular outflow tract, along with an interval increase in the size of a pericardial effusion when compared to a PET scan from five months prior (Figure 1). At the time of initial evaluation in the ED, the patient was asymptomatic from a cardiopulmonary standpoint, specifically denying chest pain or dyspnea, though endorsing chronic superficial abdominal tenderness in multiple abdominal regions. Vital signs were normal and physical examination was significant only for mild epigastric and right upper quadrant tenderness. An ECG was obtained that demonstrated sinus rhythm, low voltage, and anteroseptal myocardial injury (Figure 2).

**Figure 3:**
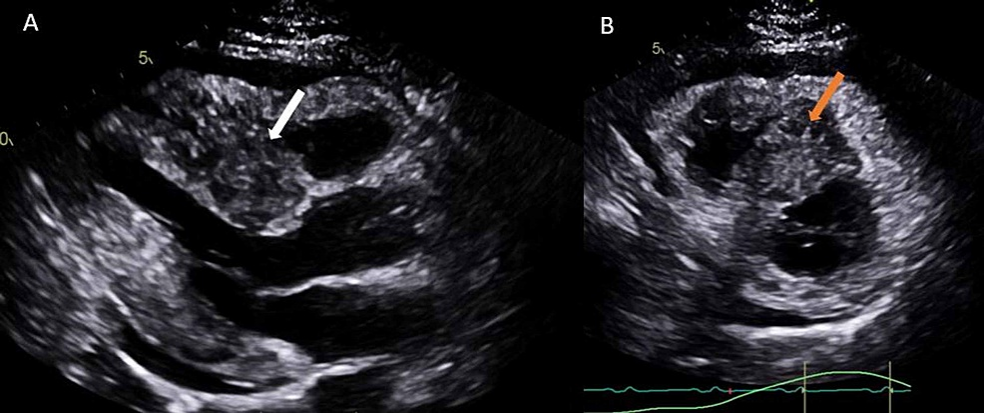
Two-dimensional TTE with parasternal long-axis view (A) showing hypoechoic mass (white arrow) in the interventricular septum and parasternal short-axis view (B) demonstrating spiculations (orange arrow) radiating out from the same central mass. TTE, transthoracic echocardiogram

In the setting of ST-segment elevation, additional concern was raised by the discovery of hypokinesis of the septal myocardium on bedside echocardiogram, though it should be noted that this area of myocardium was being impinged upon by a 47 mm × 23 mm mass in the interventricular septum (Figure 3).

However, given the lack of cardiopulmonary symptoms, the patient was treated conservatively with serial ECGs and cardiac enzymes. Initial troponin was 0.14 ng/mL (99th percentile for assay being 0.03 ng/mL) with stable values on repeat assays performed at six-hour intervals. The patient was then admitted to the Coronary Care Unit and monitored closely for signs of cardiac tamponade (i.e., pulsus paradoxus). He subsequently required pericardiocentesis for a large pericardial effusion concerning for tamponade physiology based on mitral inflow velocities. Based on cytology with negative infectious labs, the effusion was deemed secondary to his malignancy. Following a seven-day hospitalization and extensive multidisciplinary discussions with the patient and his family regarding the prognosis, the patient elected to forgo any further cardiac imaging and was discharged home with hospice, where he died shortly after discharge.

## Discussion

We present a case of characteristic ST-segment elevation suggestive of anteroseptal STEMI in a patient with no risk factors for coronary artery disease, a recently identified cardiac mass, and without any correlating symptoms. Our patient’s troponin T assay was found to be elevated above the 99th percentile of the upper reference limit on initial assessment without a subsequent rise and/or fall, and thus did not meet the criteria for myocardial infarction according to the Fourth Universal Definition of Myocardial Infarction [6]. Given that the patient was a young, active-duty male (a patient population with a low risk for atherosclerotic coronary disease making STEMI highly unlikely), and that the clinical picture was not suggestive of an acute coronary syndrome (no symptoms of myocardial ischemia), the decision was made to forgo cardiac catheterization. With the time-sensitive and life-threatening nature of STEMIs, we believe this case illustrates the importance of maintaining a suspicion for alternative diagnoses when STEMI does not fit the clinical picture as the decision to begin anticoagulation and perform a left heart catheterization can place the patient at higher risk with less potential benefit in these scenarios. After much thought and discussion, we surmised that the ECG findings were most likely attributable to a persistent current of injury related to the interventricular septal mass.

Various cases of patients with cardiac tumors presenting with ECG changes have been reported dating back to as early as 1934 [7]. We consider this case important to report because the patients in most of the previously documented cases presented with cardiopulmonary symptoms. For example, a case reported by Demir et al. in January 2019 described a 59-year-old man with a history of tongue cancer who presented to the emergency department for acute-onset chest pain [4]. The ECG demonstrated ST-segment elevation in leads V1-V6 consistent with acute anterior wall myocardial infarction and the patient was found to have a large tumor involving the right ventricular free wall on TTE. Another case published by Xiang et al. in February 2020 detailed a 66-year-old man who presented to his internist’s office with nonproductive cough, lower extremity edema, and dyspnea at rest who was found to have elevation of the ST-segment in leads II, III, and aVF, as well as leads V3-V6 concerning for acute myocardial infarction [5]. Computed tomography of the patient’s chest demonstrated anterior bulging of the left and right ventricles that was subsequently biopsied and revealed high-grade malignant spindle and epithelioid neoplasm. In contrast to these and other previously documented cases, our patient was asymptomatic from his cardiac tumor at presentation, even in the context of developing cardiac tamponade physiology on TTE.

A review of ECG characteristics of metastatic cardiac tumors presenting with ST-segment elevation published by Akgun et al. in March 2020 described particular ECG features that could potentially assist in identifying the etiology and prevent inappropriate and unnecessary interventions [8]. The authors reported that of the 36 patients for whom the ECG characteristics were evaluated, 35 (97.2%) had ST-segment elevations that corresponded to a specific coronary territory. This is consistent with the ECG findings from our patient whose pattern of injury localized to an anteroseptal distribution (i.e., left anterior descending artery), which correlated with the myocardial region where the mass was noted on echocardiogram. Interestingly, pathologic Q waves and/or poor R wave progression suggestive of infarcted myocardium were only noted in one patient in the review. The absence of these findings in our patient further supports the notion that our patient did not suffer a STEMI and, instead, the ST-segment elevation likely represented a current of injury directly related to the metastatic cardiac tumor. We were unable to further elucidate the precise pathophysiological mechanism that may have generated the current of injury as an autopsy evaluation was declined.

## Conclusions

This case report describes a young, active-duty military patient with classic findings of an anteroseptal ST-segment elevation myocardial infarction on ECG with elevated troponin level that ultimately appeared to be attributable to a current of injury from an interventricular septal mass. We consider this an important case to report as it reinforces the importance of maintaining a wide differential diagnosis for ST-segment elevation, particularly in the context of a patient with known metastatic malignancy and absence of typical anginal symptoms, risk factors, or signs of developing heart failure to help prevent unnecessary morbidity and mortality.
